# Supplementary material for: Mucosal Application of a Low-Energy Electron Inactivated Respiratory Syncytial Virus Vaccine Shows Protective Efficacy in an Animal Model
Source: Viruses. 2023 Aug 30;15(9):1846. doi: 10.3390/v15091846 (PMC10535182; doi:10.3390/v15091846)
Supplement: Supplementary file 1 [file viruses-15-01846-s001.zip › viruses-2576651-supplementary.pdf]

Supplements:

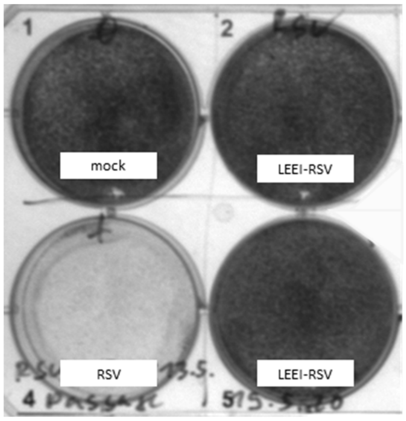

Figure S1. Inactivation of RSV by LEEI. After LEEI, the treated RSV-samples were incubated on HEp-2 cells for six days and cell culture supernatant was passaged on to fresh HEp-2 cells and incubated for another six days. Afterwards the cells were stained with crystal violet. As negative control cells were mock infected with virus-free medium or as positive control infected with untreated RSV. Shown are the plates after the second passage. In solid dark wells the cell layer was completely stained and no CPE was detected, in clear wells the cell layer was completely destroyed by viral infection.

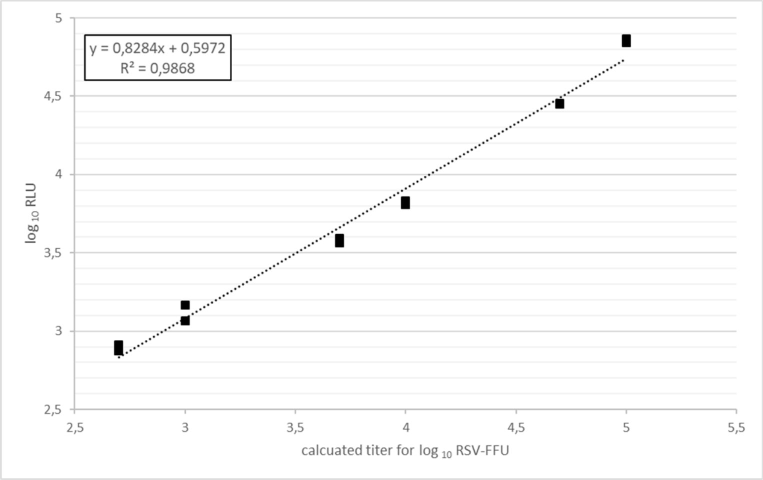

Figure S2. Standard curve for RSV amount. In order to quantify the calculated infective RSV amount of inactivated material in ELISA, a standard curve was performed in a concentration range from $1*10^5$ FFU to $5*10^2$ FFU in fivefold dilution.

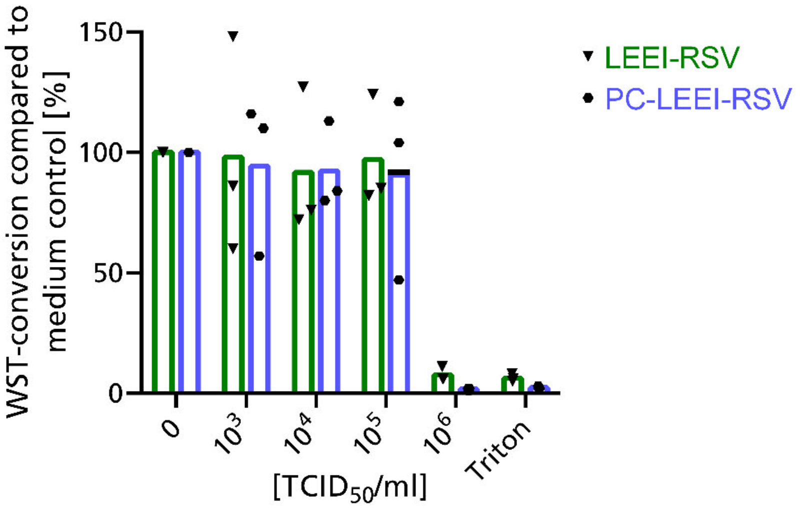

Figure S3. Metabolic activity in PCLS incubation with inactivated material. Murine PCLS were incubated with LEEI-RSV or PC-LEEI-RSV as described in Figure 2. Metabolic activity was measured with WST-1 using indicated doses of the vaccine candidates. The WST-1 conversion is shown as percentage compared to medium control (0 TCID50/ml). Each dot represents one donor and the mean for each group is indicated as bar.

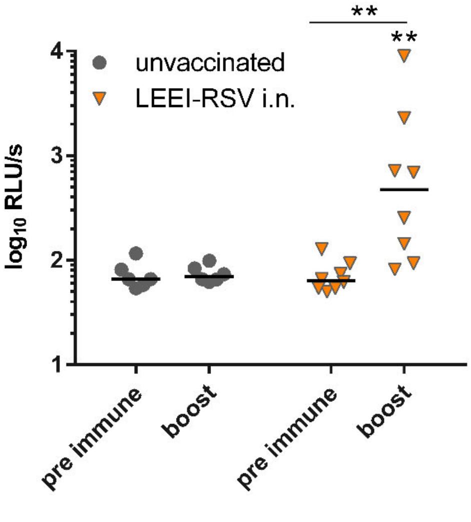

(a)

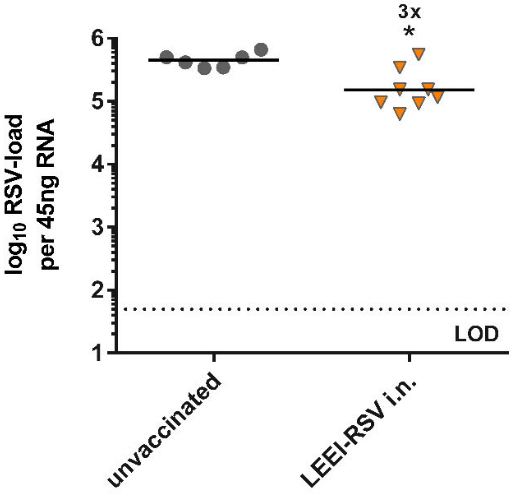

(b)

Figure S4. Systemic humoral immune response after intranasal LEEI-RSV without formulation immunized animals and viral load after RSV infection. Mice were vaccinated in a homologous prime-boost regimen with unformulated LEEI-RSV intranasal or left unvaccinated. Before (pre immune) and three weeks after prime and boost vaccination, blood samples were collected to monitor the systemic humoral immune response. Shown are serum RSV-binding IgG antibodies (a) detected by ELISA. In (a) every dot is the mean of duplicate of one animal and shown is a representative experiment out of two. Four weeks after the second immunization animals were challenged with 10^6 FFU RSV per mouse and the RSV-load in the lungs was measured five days after challenge via qRT-PCR (b). Data points represent viral copy number of each animal measured in duplicates with geometric mean of each group. Calculated viral reduction is shown in comparison to the unvaccinated control as fold reduction. Statistical evaluation was performed by Mann-Whitney test in comparison to the untreated group, or in comparison to different timepoints (line). (*: $p \leq 0.05$, **: $p \leq 0.01$) (LOD=limit of detection for viral load 50 FFU; n=6-8)
